# Do audition electives impact match success?

**DOI:** 10.3402/meo.v21.31325

**Published:** 2016-06-13

**Authors:** Elizabeth Higgins, Linnie Newman, Katherine Halligan, Margaret Miller, Sally Schwab, Lynn Kosowicz

**Affiliations:** 1 Department of Pediatrics and Internal Medicine, Albany Medical College, Albany, NY, USA; 2 Department of Medical Education, Albany Medical College, Albany, NY, USA; 3 Department of Pediatrics, Albany Medical College, Albany, NY, USA; 4 Colorado Housing and Finance Authority, Denver, CO, USA; 5 Faculty Development, New York Medical College, Valhalla, NY, USA; 6 Department of Medicine, University of Connecticut Health Center, Farmington, CT, USA

**Keywords:** audition, electives, match, 4th year students

## Abstract

**Purpose:**

The authors sought to determine the value of the audition elective to the overall success of medical students in the match.

**Method:**

The authors surveyed 1,335 fourth-year medical students at 10 medical schools in 2013. The study took place over a 2-month period immediately following the match. Medical students were emailed a 14-question survey and asked about audition electives, rank order, and cost of ‘away’ rotations.

**Results:**

One hundred percent of students wishing to match in otolaryngology, neurosurgery, plastic surgery, radiation oncology, and urology took the audition electives. The difference by specialty in the proportion of students who took an audition was statistically significant (*p*<0.001). Of the students who auditioned, 71% matched at one of their top three choices compared with 84% of non-auditioners who matched to one of their top three choices (*p*<0.01).

**Conclusions:**

Students performed a large number of ‘away’ rotations as ‘auditions’ in order to improve their chances in the match. For certain competitive specialties, virtually all students auditioned. Overall, students who did not audition were just as successful as or more successful than students who did audition.

As competition for residency spots increases, students feel more pressure to do ‘audition electives’. The term audition elective is used to describe a clinical elective taken by medical students to distinguish themselves from their peers in order to improve their chances of being selected by a residency program. The term was coined in 1987 by Swanson who was then at the Association of American Medical Colleges (AAMC) (1). At that time, audition electives were frowned upon. The AAMC committee on medical school and residency transition adopted a policy advising against excessive audition electives. Medical educators maintained that medical school should be used for broad based education rather than narrow specialization. Medical educators also argued that giving preference to audition elective students for residency positions was fundamentally discriminatory. Despite the view of medical educators, program directors appear to value the audition elective. Program directors were surveyed by the NRMP in 2014 and asked to rate the relative importance of factors in selecting students for interviews and for ranking them in the match (2). Program directors consistently rated the audition elective as important in both the interview and ranking process. For some specialties, it was the most important factor. Voght et al. reviewed students from 1977 to 1997 and found no statistically significant difference between students who auditioned and those who did not in their success in the match (3). In 1995, Fabri et al. investigated the outcome of 99 students who took audition electives in surgery. They found that although the elective increased the probability of an interview, it did not alter the probability of a successful match and may have actually decreased competitiveness for the general surgery applicant (4). Crane et al. analyzed a survey of 118 emergency medicine program directors in 2000 and showed that audition electives were of moderate importance, ranking as less important than emergency medicine rotation grade, interview, clinical grades, or letters of recommendation, but more important than both United States Medical Licensing Examination (USMLE) I and II scores (5). Bernstein et al. showed in 2002 through a survey of 156 orthopedic surgery program directors that residency program directors consider audition electives at their respective institutions equally as important as USMLE I scores and class rank in selecting applicants (6). In 2010, Huggett et al. confirmed that audition electives were widely used but poorly understood and called for further research to examine outcomes at the individual student level (7).

We aimed to answer the question: Are audition electives associated with medical students’ overall success in the match and does the value of the elective depend on the specialty?

## Method

A 14-question survey was emailed to 1,335 fourth-year medical students at 10 medical schools after the match of 2013 (List 1). Participation was voluntary, and all responses were anonymous.

## Participants

Participating schools were chosen based on interest demonstrated at a discussion group at the National AAMC Meeting in 2012. The following schools comprised the sample for this study: Albany Medical College, Albert Einstein College of Medicine, George Washington School of Medicine, New York Medical College, University of Alabama School of Medicine, University of California Davis School of Medicine, University of Central Florida, University of Connecticut School of Medicine, University of Missouri-Kansas City School of Medicine, and University of Texas Medical Branch School of Medicine.

## Measures

Identical surveys were created for each school. To establish content validity, the development of the survey instrument was informed by the literature (7). The survey instrument was pilot tested in 2008–2011 and changes were made based on feedback and informal cognitive interviews with students. Anchors were provided for the first 12 questions. The open-ended questions were not analyzed.

## Procedure

The Albany Medical College Institutional Review Board approved all components of this study. Prospective participants were contacted via email by a local ‘champion’ at the participating school. Informed consent was not required by the institutional review board and participation was voluntary. No incentives were provided to study participants. Surveys were emailed three times to increase response rates. Total survey time was 2 months. Mantel–Haenszel chi-square statistics were performed and conventional values (*p*<0.05 or greater) were used for statistical significance. For 2×2 tables, the Mantel–Haenszel Chi-square statistic was used. For larger dimensions, the Pearson Chi-square statistic was used. The statistical software package was SPSS version 19.

## Results

Of the 1,335 students surveyed, 756 responded (57%). Of these, 474 (63%) took one or more audition electives. One hundred percent of students wishing to match in otolaryngology, neurosurgery, plastic surgery, radiation oncology, and urology auditioned. No students wishing to match in pathology took an audition elective. The difference by specialty in the proportion of students who took an audition was statistically significant: Chi-square for proportion by specialty was Pearson Chi-square (20)=133.2, *p*<0.0001 and Mantel–Haenszel chi-square (1)=16.91, *p*<0.001. Follow-up examination of standardized residuals indicated specialties with significantly different percentages of auditions. Table 1 displays the audition rates by specialty type.

**Table 1:** Percentage of students who matched in Audition Elective Survey Results 2013: percentage of students who auditioned in each specialty

90–100%	50–75%	<50%
Dermatology^a^	General surgery	Anesthesia^a^
Emergency medicine^a^	Neurology	Family medicine
Neurosurgery	OB/GYN	Internal medicine^a^
Otolaryngology	Pathology	Med/peds
Ophthalmology	Pediatrics	Psychiatry
Orthopedics^a^	Radiology	
Plastic surgery^a^	PM&R (83%)	
Radiation oncology		
Urology^a^		

a Indicates significantly higher or lower percentage of audition rotations (*p*<0.001).

Overall, 93.9% (710/756) of students in this survey matched in the specialty of their choice. Of the students who auditioned, 49% (231/474) matched at their number one choice and 52% (147/282) of non-auditioners matched at their number one choice. This difference is not statistically significant. Seventy-one percent (337/474) of auditioners matched at one of their top three choices compared with 84% (236/282) of non-auditioners who matched to one of their top three choices. This difference is statistically significant at *p*<0.01. The odds ratio of students not choosing an audition elective and still matching in one of their top three choices is 2.181 (95% confidence interval 1.495, 3.181).

Students reported that they ranked a program lower because of their audition. Fifty-three percent (251/474) (Pearson Chi-square (20)=41.09, *p*=0.004) reported that they ranked a program lower on their list or not at all after their audition elective because of information they had learned while on their rotation. Overall, 39% (185/474) of students who auditioned matched where they performed their audition. Table 2 illustrates the percentage of students who matched at their audition by specialty. Results ranged from 80% in radiation oncology to 3% in general surgery. Percent match by audition was not statistically significant; however, this may be due to small sample sizes per specialty.

**Table 2:** Audition Elective Survey Results 2013: specialty-specific data regarding an audition and the match

	Applied in specialty	Auditioned in specialty	% matched at audition (*n*)
Radiation oncology	5	5	80 (4)
PM&R	6	5	60 (3)
Orthopedics	33	31	58 (18)
Plastic surgery	7	7	57 (4)
Pediatrics	120	65	49 (32)
Psychiatry	35	16	44 (7)
Anesthesia	47	17	41 (7)
Med/peds	12	5	40 (2)
Internal medicine	150	69	39 (27)
Neurosurgery	6	6	33 (2)
Urology	11	11	36 (4)
Emergency medicine	59	56	32 (18)
Ophthalmology	19	17	35 (6)
Otolaryngology	14	12	33 (4)
Neurology	15	10	30 (3)
Family medicine	50	24	29 (7)
Radiology	46	28	28 (8)
OB/GYN	41	30	23 (7)
Dermatology	20	18	17 (3)
General surgery	44	33	3 (1)
Pathology	9	5	0
Prelim medicine only	3	0	0

Students who chose not to audition cited cost most frequently as the reason they did not select an audition elective. Students reported that the cost of ‘away’ rotations ranged from $500 to more than $2,000.

## Discussion

Competition for residency slots is increasing. In 2015, 18,025 US allopathic medical seniors (US seniors) were among 41,334 registrants in the match competing for 27,293 PGY1 spots (8). Much to the chagrin of medical educators, the pursuit of a residency position, otherwise known as the ‘pre-residency’ syndrome (9), occupies much of the fourth-year schedule for medical students. A recent study quoted in the New England Journal of Medicine (NEJM) showed that 56% of students complete at least one audition elective, and this varies greatly by specialty (10). They found that the majority (66%) did not match at the location of their ‘away’ rotation. Our results demonstrated that nearly 100% of students are auditioning for certain specialties (dermatology, emergency medicine, otolaryngology, neurosurgery, ophthalmology, orthopedic surgery, plastic surgery, radiation oncology, and urology) and many audition at several institutions.

The students in our survey had an overall match rate of 93.9%. This is comparable with the 95.7% rate for all US seniors in the National Resident Matching Program. In 2015, 78.4% of US seniors matched to one of their top three choices. The students in our survey who auditioned had a lower match rate to their top three choices (71%) compared with non-auditioners (84%). It is possible that this is due to the fact that they were applying to more competitive specialties. It is also possible that their performance was suboptimal or they were not competitive for the residency programs for which they applied based on their transcript, AOA status, or USMLE scores.

For some competitive specialties, our results suggested that the audition electives are valuable. For example, students applying for radiation oncology were very likely to match at their audition rotation. Four out of five students matched at their audition program. The small sample size limits the ability to calculate statistical significance. Audition rotations in surgery, however, appeared to have a negative influence. Students interested in surgery do not match at their audition elective. Only one student out of 33 matched at an audition rotation in general surgery. We wonder if this is due to what we call the ‘dirty laundry effect’. Overall, 53% of the 474 students who auditioned ranked programs lower due to information they learned (‘dirty laundry’) while on the rotation. Our study has several limitations. Although our overall response rate was high (57%), response rates among the 10 schools varied from 18 to 90%. The timing of the survey directly after the match and prior to graduation left a very short time for completion. Another limitation of our study was the small sample size for some specialties, but this is consistent with the number of people entering these specialties. We suggest a longer period of time for future studies, so as to include multiple years of students, as a direction for future research. A final limitation is that we did not ask about grades or step scores. These factors are significant variables in the match.

Students need guidance on the value of audition electives specifically as they relate to their specialty of choice. Some faculty advice students to take ‘away’ electives to demonstrate that they can succeed in an environment other than that of their ‘home school’. Students sacrifice time from their fourth-year curriculum and spend a significant amount of money on audition electives. This study will serve to help faculty advise students about the value of these electives. For example, students entering general surgery should be advised that an audition elective may not be necessary. Further research is needed to examine the necessity of audition rotations, especially in specialties where the number of auditions is high but the match rate is low (dermatology and general surgery). The answers to questions 13 and 14 of our survey instrument yielded information that may be analyzed for a future paper and may help to understand the student perspectives of the impact and experience of the audition rotation.

We wonder about the unintended negative consequences of the use of the electronic Visiting Student Application Service, VSAS, which is an AAMC application designed to streamline the application process for senior ‘away’ electives at LCME approved medical schools and independent academic medical centers. This service allows students to submit just one application for all institutions and was created at the request of two GSA committees: Committee on Student Records (COSR) and Committee on Student Affairs (COSA) (11). It has facilitated students to apply for audition electives essentially creating a ‘prematch application’. This phenomenon and the policies and procedures of individual programs regarding visiting students deserve further investigation.

Finally, we propose that advisors and deans consider several things as they advise fourth-year students with regard to audition electives. First, many students who did not perform audition electives still matched in their specialty of choice. For example, most students who applied in pediatrics did not audition and of those who did audition, less than half matched at a program where they did an ‘away’ rotation. Therefore, advisors should be clear that auditions are not necessary for all students. Clearly, the necessity of auditions is specialty specific. Also, some excellent students may not perform well on an audition elective due to multiple factors and hence this may negatively impact their match. Advisors who know their students well can address this on a case by case basis. Finally, although there is significant pressure on students to do ‘away’ rotations, students need to consider cost, schedules, and personality fit before deciding to take ‘away’ auditions.

## Previous presentations

Northeastern Group on Educational Affairs, 2014 Regional Meeting, New Haven, Connecticut.

## References

[1] Halperin E (1988). The audition elective. Int J Radiat Oncol Biol Phys.

[2] National Resident Matching Program (2014). Data release and research committee: results of the 2014 NRMP program director survey.

[3] Voght BH, Thanel FH, Hearns VL (2000). The audition elective and its relation to success in the National Residency Matching Program. Teach Learn Med.

[4] Fabri PJ, Powell DL, Cupps NB (1995). Is there value in audition extramurals?. Am J Surg.

[5] Crane JT, Ferraro CM (2000). Selection criteria for emergency medicine residency applicants. Acad Emerg Med.

[6] Bernstein AD, Jazrawi LM, Elbeshbeshy B, Della Valle CJ, Zuckerman JD (2002–2003). An analysis of orthopaedic residency selection criteria. Bull Hosp Jt Dis.

[7] Huggett KN, Borges NJ, Jeffries WB, Lofgreen AS (2010). Audition electives: do audition electives improve competitiveness in the national matching program?. Ann Behav Sci Med Educ.

[8] The MATCH: results and data of the 2014 residency match.

[9] Swanson AG (1985). The preresidency syndrome: an incipient epidemic of educational disruption. J Med Educ.

[10] Aagaard EM, Abazz M (2016). The residency application process-burden and consequences. New Engl J Med.

[11] VSAS website https://www.aamc.org/students/medstudents/vsas/.

